# Educational interventions on breast cancer in men and women: a necessity in primary healthcare

**DOI:** 10.3332/ecancer.2021.1255

**Published:** 2021-06-22

**Authors:** Ortega Jiménez Mayra del Carmen, García Rodríguez Deysi Emilia, Brenda Hidalgo Mares, Ortega Jiménez Marcela

**Affiliations:** 1 Department of Nursing and Obstetrics, University of Guanajuato, Celaya, CP38110, Mexico; 2 Doctoral Studies, Universidad Internacional Iberoamericana (UNINI-Mexico), Campeche, CP 24560 Mexico; 3 Universidad Internacional Iberoamericana (UNINI-Mexico), Campeche, CP 24560, Mexico; 4 Department of Nursing and Obstetrics, University of Guanajuato, Celaya CP38110, Mexico

**Keywords:** breast cancer, intervention education, prevention, primary

## Abstract

Breast cancer (BC) is one of the most common diseases in the global population. It most commonly presents in women; however, there has been an increase in the number of men diagnosed with the disease, although at a lower rate. Its specific characteristics and associated risk factors mean that preventative measures are considered to be one of the most important methods of avoiding BC. Therefore, education is a fundamental part of this process. The objective of this study is to report on the educational interventions on BC carried out in healthcare between 2016 and 2021. To this end, an integrative review was carried out using the following databases: PubMed (NCBI), Science Direct, Scopus, SciELO and Google Scholar, using the keywords ‘breast cancer’, ‘intervention education’, ‘prevention’ and the Boolean operator ‘AND’. Quantitative, full-text articles in English, Spanish or Portuguese were included. Finally, 19 articles were selected for analysis and it was found that, with regard to educational interventions on BC carried out in healthcare, one article included men and women and the remaining 18 included only women, with interventions carried out in sessions, workshops, in stages and using dynamic techniques. Therefore, there is a pressing need for educational interventions on BC for men and women at all stages of life; however, priority should be given to the young population in order to allow for early prevention. These interventions do not generate costs for the health sector, but they have a positive effect by increasing knowledge and promoting self-care.

## Introduction

Breast cancer (BC) is a malignant disease where the rapid, abnormal and uncontrolled spread of cells in the different tissue types found in the mammary gland, such as epithelial cells of the mammary ducts or lobules, form a tumour that invades neighbouring tissues and metastasises to other organs in the body [1].

A global study found that these diseases cause a series of economic, social and family-related issues for those suffering from chronic degenerative diseases such as BC. It has one of the highest mortality rates for those suffering from this particular disease, and it is the second most commonly diagnosed cancer globally and the most common cancer among women [2].

During a comprehensive global study, a prediction of the incidence of BC was made and it was determined that there will be approximately 3.2 million new cases by 2050 [3]. This will mainly affect the least developed regions of the world where 56% of new cases and 63% of deaths are reported [4]. It is estimated that women who reach 85 years of age or older will have a 1 in 9 chance of developing BC [5].

In Mexico, BC is among the three most prevalent types of cancer. It mainly affects women, and men suffer to a lesser extent. Men are at a disadvantage due to the methods used to disseminate information on the topic which tends to focus on the results of BC in women as they are more likely to develop the disease. This can lead to men believing that they cannot suffer from this type of cancer [6].

Every day, women are affected more and more by BC due to an apparent lack of preventative preparation and a lack of urgency to perform breast self-examinations at an early age. This is due to insufficient guidance that is offered to both genders on this issue. Breast carcinoma is rare before the age of 20, but from this age the incidence rises steadily [7].

In addition, males with BC represents between 0.5% and 1% of all BC cases diagnosed worldwide, with a male to female ratio of 1:100. Even though rates have increased over the last 25 years, it generally has a poor prognosis with a poor survival rate due to the fact that most cases are diagnosed in very late stages [8]. Miao *et al* [9] estimated the incidence in men to be 0.40 in 100,000 people/year.

There has been a greater emphasis placed on the importance of early BC detection through health promotion, with the aim of reducing morbidity and mortality rates at both international and national levels. However, this has not had a strong enough impact on the population, so more effective guidance on prevention is required. One of the main problems is the lack of knowledge as well as sociocultural factors, applicable to both men and women.

Mexico’s short- and medium-term outlook regarding BC is not encouraging because the programmes that were created and implemented for prevention and early detection have not adequately curbed the number of cases and deaths from these diseases [10].

Therefore, there is a clear need for effective prevention work to be developed in the population, targeting both genders and incorporating young people, with the overarching aim of raising awareness of the importance of breast care.

BC diagnoses are rare among women over the age of 30, however, the development of cancer even where rates are very low can hold great significance because when it does develop, both diagnosis and treatment significantly affect quality of life [11]. This can result in premature menopause; infertility; osteoporosis; weight gain; common psychological effects such as changes to body image, cognitive function and the constant threat of recurrence and early death; as well as social effects relating to work, relationships with partners, caring for loved ones and dealing with stigma [12]. That is why interventions should be aimed at those in all stages of life.

Furthermore, it has been determined that women who live in countries with a high socioeconomic level have a higher risk of developing BC, but there is a higher risk of death among women who live in poor countries, due to the fact that they have less access to healthcare services for early detection, treatment and control of the disease [13].

Although people claim to be knowledgeable about BC, it has been reported that people find it difficult to identify risk factors and prevention strategies, as well as specific information regarding treatment. Thus, it is necessary to improve education on preventing this type of cancer in study programmes and to encourage health promotion [14].

Understandably, the healthcare system plays a key role and it must guarantee access to services without discriminating on the basis of socioeconomic conditions, thus ensuring equal access to opportunities and quality provision of preventative, diagnostic and therapeutic services, to decrease the mortality of preventable cancers in women attributed to diagnosis and timely treatment. This can facilitate early detection and timely treatment [15].

Early detection of BC has been shown to reduce mortality and is an important step in reducing the burden of this disease on the public healthcare system. However, despite the infrastructure and availability of organised screening programmes, participation in screenings for many types of cancer remains below established targets [16].

In this regard, achieving a reduction in modifiable risk factors related to the development of BC is highly significant, and this involves promoting healthy lifestyles (HL). The fundamental aspects of this are adopting a healthy diet, controlling body weight, engaging in physical activity, limiting alcohol con sumption and not smoking. Adopting a healthier lifestyle results in a 17%–58% decrease in BC risk factors and significantly reduces the risk of cancer morbidity and mortality, and should therefore be prioritised in the prevention of BC [17].

Poor nutrition and insufficient exercise are significant factors in the development of BC, and are related to lifestyle (diets high in carbohydrates and both animal and trans fats but low in fibre, as well as obesity and sedentary behaviour) [18]. In Latin America, there exists a common risk and epidemiological profile due to the high frequency of some risk factors, such as excess weight, obesity, low breastfeeding rates, little physical activity and hormone use. It also refers to the lack of health information, which is reflected in limited access to primary and secondary prevention services [19].

Another modifiable risk factor is associated with tobacco use. Four thousand chemicals have been found in oral tobacco products, 60 of which are considered carcinogens [20].

Additionally, alcohol consumption is also considered to be a modifiable risk factor and is associated with an increased risk of BC, because there is a linear dose–response relationship between alcohol consumption, risk and this type of cancer [21].

Another modifiable risk factor is the tendency not to undertake self-examination, as indicated in a study conducted in Lima, Peru, which reports that 62.5% of women do not know the frequency with which self-examination should be undertaken and 78.8% is not aware of its usefulness for early detection of cancer [22].

Breast self-examination should be performed by all women from the age of 20, between the 5th and 7th day, after the menstrual cycle, since this is when the breasts are softer. Women who no longer have their period will have to do it on a specific day of each month, this is to create a habit and try to improve the detection technique [23].

The risk factors found suggest that it would be beneficial to develop actions that promote HL [24]. Thus, it should be oriented at all stages of a person’s life, and in particular it should be started in early childhood to encourage them to take responsibility for self-care of health, to reduce risk factors where possible and promote HL. In addition, promoting health in order to detect BC should include self-examination, clinical examination and mammography.

It is based on the assumption that the main ways to foster understanding about the prevention and characteristics of BC are through educational measures, through teaching in a friendly way to this community and at a low cost; which can have a positive impact on the early identification of cancer.

Studies associated with the effectiveness of measures carried out in this area show that educational interventions for the prevention and early detection of BC in women of childbearing age are effective, and that more than 60% of women gained sufficient knowledge about the prevention and early detection of BC [25].

On the other hand, in a study carried out in Mexico City that had the objective of assessing the impact of the syllabus on modifying behaviour and attitudes related to BC in teenagers, the results showed that the programme was successful, since the sessions included in the programme were adequate for improving the adolescents’ level of knowledge about BC [26].

Educational interventions have been recommended as an effective public health approach in the comprehensive control of BC, especially among young people.

At the same time, the importance of preventive BC health education interventions, in all stages of life, is reiterated, in order to develop health-promoting behaviours that allow the detection and timely control of risks in the development of BC. Its effectiveness has been demonstrated, but the need to have the knowledge, skills and competencies necessary to be able to transmit relevant and updated education on the subject is highlighted [27].

That is why a review of the bibliography referring to health interventions in BC in men and women at any stage of their lives has been undertaken, in order to show the work done in this regard, highlighting its need and importance for society as a whole. This enhances the possibility of adopting, adapting and improving it, in order to develop a primary prevention programme in effective health associated with this pathology.

**Approach**

For the development of the study, a search of scientific documents was carried out in databases PubMed (NCBI ), SciELO, Scopus and the Google Scholar search engine, using the descriptors ‘breast cancer’, ‘intervention education’, ‘prevention’, ‘primary’ and the Boolean operators ‘AND’.

The articles included quantitative and qualitative studies, full texts, written in English, Spanish or Portuguese, published from 2016 to 2021.

For the inclusion criteria, to be considered articles needed to be full-text, published in the aforementioned databases and in the search engine, products of primary research carried out in English, Spanish or Portuguese where the concept of educational interventions in BC is addressed, focused in primary prevention applied to men, women or both in all stages of life, with the publication date 2016–2021 and based on quantitative, qualitative or mixed-focus studies.

**Figure 1.**
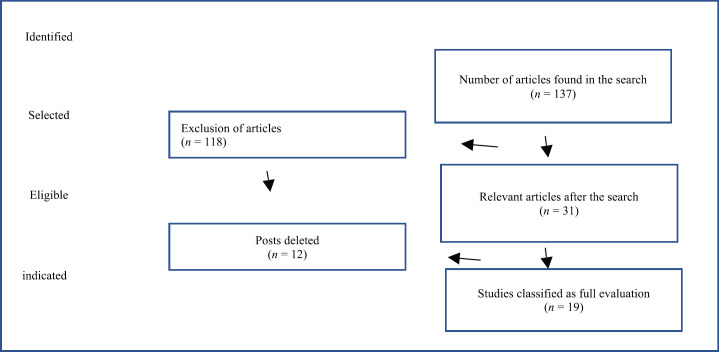
Flowchart for educational interventions in BC.

During the period from 2019 to 2020, an exhaustive search was made in the scientific literature for articles describing educational interventions in BC, through which 137 articles were found; with 19 articles remaining to be analysed (Figure 1).

## Results

In the present literature review, predominantly articles in English and Spanish were used, finding the following; eleven in PubMed, five in SciELO, zero in Scopus and three in the Google Scholar search engine, obtaining a total of 19 selected for the development of this bibliographic review.

Of the 19 articles, all of them on educational interventions of BC, one included men and women and the remaining 18 included women only.

Of these articles found, the types of study in the order of predominance were: those of an experimental cut from a quantitative methodology. Of these, six of the selected papers are classified as quasi-experimental; one responds to an experimental design; two claim to be pre-experimental studies; two are trials. In addition, a pilot test, a descriptive and a prospective study were carried out. Only three of them were classified as mixed studies, and in two this fact is not mentioned.

Amongst the main topics addressed, we find a predominance of work aimed at promoting, in some way, knowledge about BC, especially linked to self-examination and the prevention of risk factors. Other studies, in order of appearance, respond to awareness/education work. To a lesser extent, there are more specific approaches linked to beliefs, attitudes, behaviours. All studies, however, are aimed at prevention, and it is important to note that in one of them they use technologies as tools to mediate the process of learning.

The countries most represented in these studies are: Colombia, in four; Mexico and Cuba in two studies each. Next, countries that appear in one study such as Peru, Saudi Arabia, Malaysia, Iran, India, Spain Brazil, Ethiopia and England, and two studies that do not contextualise their results. The largest scientific production/publication is associated with the years 2019–2020, with 10 of the publications concentrated in that period.

We observe that 13 of the studies consider interventions in the population of adolescents and young people, whilst 2 of them include also those over 20 years old (up to 60); 5 of them focus only on adults and 1 does not specify the data.

On the other hand, it is observed that, in the studies presented, there is a tendency to use educational strategies where information/discussion/practical exemplification is weighted, rather than active participation in the preventive educational process.

The summary of the particularities of the studies found on educational interventions in BC is presented in Table 1.

## Discussion

Within the studies found, health education is addressed in a general sense, being considered as a dynamic and continuous process that includes self-care behaviours, compliance with healthcare requirements, recommendations, satisfaction in caring for health and adjustments in the person’s quality of life [28].

Prevention activities include educational communication with the public to create awareness of risk factors and to promote HL which can contribute to reducing BC morbidity, as well as promoting demand for early detection in order to improve opportunities for diagnosis and treatment [29, 30]. Furthermore, consideration is given to the need to establish BC detection strategies and define methods to improve prevention based on educational intervention [31], as well as preventative methods for modifiable risk factors.

One of the modifiable risk factors most often cited in this bibliographical review is regular breast examination. The tendency cited is towards nonexistent or inadequate self-examination. It is noted that women are unaware of how often self-examination should be done or of its usefulness in early detection of cancer [32, 33]. It is important to emphasise the need for breast self-examination within a community and to make that need visible, as a means of promoting women’s health and as a primary method of preventing BC. This practice needs to be one of the priorities within primary healthcare [34, 35].

The strategies which have been provided at international and national levels in terms of prevention and promotion of health include: breast examination, mammography and early detection [36].

In this context, it was found that interventions addressing awareness, attitudes and practices relating to breast self-examination in adolescent women provide positive results [34], other studies in young people state that breast self-examination should be carried out from the age of 20 onwards. However, a percentage of the population surveyed is unaware of this and there is considerable ignorance about the age at which breast examination should start [32]. Other studies in young people mention that those who managed to acquire a level of awareness of risk factors and BC did not put this into practice in their daily life [28, 37–39].

With respect to people’s knowledge of and attitudes towards BC, they indicate that these vary depending on age and marital status, with a pronounced lack of awareness of preventive measures and risk factors for BC among young and single people [5, 40].

Another noteworthy element in relation to breast self-examination is failure to take the menstrual cycle into account, as a factor for consideration in good practice, which indicates a persistent failing in this aspect, showing the need to educate all age groups [41].

It was found that in interventions in the 30–65 years age range, there is insufficient knowledge about BC. However, after the educational intervention, there was generally a very good level and following the intervention no women were found to have a poor level of knowledge [42, 43].

Mention was made of the need to implement health promotion and education strategies to tackle non-transmissible diseases and other health conditions. Particular emphasis was placed on working to reduce premature mortality due to BC. In this respect, it is essential to address BC from an epidemiological-preventive and social perspective, instead of merely placing it within a curative care paradigm [44].

Several studies have been carried out in which education and health are structured as connecting axes in preventing the appearance of BC, including basic information on cancer facts and figures, BC epidemiology, risk factors for developing cancer, signs and symptoms, etc., [28, 45].

After analysing the data, it can be said that the level of awareness in individuals who participated in the studies contained in the research shown increased significantly [43] as a result of the various educational interventions on BC, particularly in aspects concerning new information and early detection methods. This indicates that the educational interventions are effective, managing to significantly increase the level of awareness in all their variants, leading to future quality of life through timely detection, linked with prevention and control of the disease and with improvements in life styles [33, 37, 46–60].

These results corroborate the need to develop policies that guide the development of information, education and counselling programmes in order to strengthen awareness of risk factors, and the signs and symptoms that require immediate health care, in connection with BC [36, 38].

It is therefore necessary to design new teaching methods based on these educational interventions in order to increase the efficiency of the teaching/learning process in health education, and its relationship with BC, since the government has not so far managed to make an impact in early detection of this condition [34, 61, 62].

In this light, educational initiatives have been recommended as effective public measures for the integrated control of BC, particularly among young people [28, 33, 37].

It should be stressed that lack of health education is a predisposing factor that prevents women and men of all ages from obtaining precise information about what they need to do to prevent BC, and to detect it at an early stage. Self-examination of the breasts is considered one of the core aspects of such education, as it enables timely detection of changes such as benign nodules, cysts, etc.

In the context of these searches, there is evidence about educational interventions to prevent BC in the under 20s, so emphasis is placed on the need to ensure successful intervention from an early age [28, 47].

On the other hand, it can be seen that the male population barely participates in this learning process, demonstrating the urgent need to develop educational interventions that include not only women, but also men, since according to these figures 98% of cases in males are diagnosed at an advanced stage of the disease, worsening their prognosis compared to women [63]. For example, in the present study, only one research project was found that covered both sexes.

It is therefore necessary for educational programmes to be designed and implemented with an emphasis on a change in attitudes towards health promotion [42].

It is recommended that providers of information on BC make use of educational interventions to reduce mortalities and promote women’s health [64]. It will be necessary to expand the intervention to more people and other health areas in order to more precisely demonstrate its reliability. It would also be advisable to make a technical assessment of the viability of interventions that have been designed, and measure their impact on early diagnosis of this type of cancer [28].

Certain characteristics have been identified which increase the effectiveness of educational interventions. These findings points to the development of programmes designed with integrated interventions. These should include aspects linked to quality of life, social and family support. The may be periodic or include follow-up over time, mix teaching approaches, make use of those that involve greater participation by attendees. They will need to be assessed using experimental methods, validated and reliable instruments that take into account process indicators, and quality of care results from attendees [28, 65].

## Conclusions

BC education interventions are effective, low-cost strategies, which can encourage their implementation in health policies. Nevertheless, they should be addressed to men and women, at all stages of life, encouraging self-care through prevention and health promotion. They should look at dietary factors, physical activity, Body Mass Index, alcohol consumption, hormonal factors, breast self-examination, thus helping to develop HL. Although incidence is very low in males compared to females, the diagnosis has a higher mortality rate due to late detection. All of this calls for multidisciplinary health interventions, not forgetting that in order to achieve the desired impact, there needs to be long-term follow-up, to verify whether the aims of the intervention are being met.

## Conflicts of interest and funding

Funded by the University of Guanajuato.

## Figures and Tables

**Table 1.** Revised articles on educational interventions in BC.

No.	Reference	Database	Title	Target	Study type	Sample	Study population	Type and number of sessions
1.	Bernal B, Figueroa D, Gutierrez J, Ramirez R, Carriño J y Castellanos A (2017)	Google Scholar	Perception and knowledge of breast self-examination before and after an intervention Health education for adolescents in Tunja	Perception and knowledge of breast self-examination before and after an educational health intervention in adolescents of Tunja	Mixed	Availability	Females aged 14–19	A Question-based dynamics of health education
2.	Masso AM, Meneses JF, Correa JE, Tovar A, Alba PA and Charry CE (2018)	PubMed	Effects of an educational intervention on breast self-examination, knowledge related to breast cancer prevention and healthy lifestyles in school children in a low-income area of Bogotá, Colombia	To evaluate the effects of an educational intervention in the practice of breast self-examination, knowledge and practice of healthy lifestyles for the prevention of breast cancer in female students of a public school in the town of Ciudad Bolívar in Bogotá, Colombia	Does not mention	Availability	155 adolescent women (10–20 years)	Two educational sessions of 90 minutes each The contents of the intervention were integrated into the school curriculum (videos, presentations and discussions)
3.	Alameer A, Mahfouz MS, Alamir Y, Ali N and Darraj A (2019)	PubMed	Effect of Health Education on Female Teachers’ Knowledge and Practices Regarding Early Breast Cancer Detection and Screening in Jazan Area: a Quasi-Experimental Study	Evaluation of the effectiveness of health education in the improvement of knowledge and practices of primary school teachers with respect to detection tools and early detection of breast cancer	Quasi-experimental	Randomised	150 primary school teachers (75 in the control group, 75 in the intervention group)	Meeting, PowerPoint presentation and practical session (60 minutes each)
4.	Akhtari M, Juni MH, Said SM, Ismail IZ, Latiff LA and Ataollahi S (2016)	PubMed	Result of a randomized control trial to increase breast health awareness among young females in Malaysia	The development, implementation and evaluation of the effectiveness of the breast health awareness programme, based on health beliefs surrounding knowledge of breast cancer and breast self-examination and the practice of breast self-examination for female Malaysian students	Randomised controlled trial	Randomised	370 students	16 workshops lasting 2 hours
5.	Kissal A, Kartal B (2019)	PubMed	Effects of Health Belief Model-Based Education on Health Beliefs and Breast Self-Examination in Nursing Students	Investigation of the effect of an education programme based on a model of health beliefs (HB) of nursing students and their practices of breast self-examination (BSE)	Semi-experimental intervention with single group, pre and post-test design	Not recorded	48 students	Use of leaflets as educational material Time not referenced
6.	Termeh Zonouzy, V., Niknami, S., Ghofranipo ur, F., y Montazeri, A. (2018).	PubMed	An educational intervention based on the extended parallel process model to improve attitude, behavioral intention, and early breast cancer diagnosis: a randomized trial	Assessment of the effectiveness of an educational intervention based fear appeals using the extended parallel process model (EPPM) to improve attitudes, intention and early breast cancer diagnosis in Iranian women	Cluster-randomised trial with two parallel groups	Randomised	438 women, 40 years old and above	Use of leaflets as educational material Time not referenced
7.	Nisha B and Murali R (2020)	PubMed	Impact of Health Education Intervention on Breast Cancer Awareness among Rural Women of Tamil Nadu	Assessment of the impact of a health education interventional programme on breast health awareness and BSE among rural women of Tamil Nadu	Quasi-experimental	Randomised	266 women (aged 20–60 years)	Interactive sessions, PowerPoint presentations, stories Time not referenced
8.	Pons-Rodriguez A, Martínez M, Perestelo L, Garcia M, Sala M, Rué M and the InforMa Group (2020)	PubMed	Informed choice in breast cancer screening: the role of education	Evaluation of the effect of receiving information about the benefits and harms of breast cancer screening in informed choice, according to educational level	Experimental	Randomised	400 women (aged 49–50 years)	Standard leaflet that recommended participating in the screening programme (control group) a decision aid tool consisting an informative brochure (intervention group) Time not referenced
9.	Pereira A, Destro JR, Picinin M, Garcia LF and Rodrigues TF (2020)	PubMed	Effects of an WhatsApp-Delivered Education Intervention to Enhance Breast Cancer Knowledge in Women: Mixed-Methods Study	Analysis of the potential of WhatsApp as a health education tool used to improve women’s knowledge on the risk reduction of breast cancer. Aimed to understand how women feel sensitised within the WhatsApp group throughout the intervention and how they incorporate information posted to improve knowledge about early detection and risk reduction methods	Mixed-methods	Not recorded	35 women (between 45 and 69 years old)	3 weeks after starting to use WhatsApp as a health education tool
10.	Abera H, Mengistu D and Bedaso A (2017)	PubMed	Effectiveness of planned teaching intervention on knowledge and practice of breast self-examination among first year midwifery students	The aim of the study is to assess the effectiveness of planned teaching programme on knowledge and practice of breast self-examination among first year midwifery students in Hawassa Health Sciences College	Pre-experimental	Randomised	61 students aged 20 ± 2 years	Theoretical (module) and practical sessions based around knowledge and practice such as conferences, audiovisual and practical demonstrations
11.	Soto-Perez E, Smith DD, Rojo MP, Hurria A, Pavas AM, Gitler R, Mohar A and Chavarri Y (2017)	PubMed	Implementation of a School-Based Educational Program to Increase Breast Cancer Awareness and Promote Intergenerational Transmission of Knowledge in a Rural Mexican Community	We tested the feasibility of implementing a school-based breast cancer educational programme for adolescents in a rural Mexican community	Pilot study	Not recorded	114 adolescent students	5 information and discussion sessions (40 minutes)
12.	Omrani A, Wakefield J, Smith J, Wadey R and Brown N (2020)	PubMed	Breast Education Improves Adolescent Girls’ Breast Knowledge, Attitudes to Breasts and Engagement with Positive Breast Habits	Evaluation of the short- and longer-term impact of a 50 minute breast education intervention on adolescent girls’ (11–14 years) breast knowledge, attitudes to breasts and engagement with positive breast habits	A mixed methods, controlled, longitudinal, cohort design, using two control schools (n: 412; receiving no intervention) and two intervention schools (n: 375; receiving the intervention)	Availability	787 students (11–14 years)	One 50 minute session consisting discussion groups and a PowerPoint slideshow presentation
13.	Hernández I, González Y, Heredia L, Heredia A, Conde M and Aguilar S (2011)	SciELO	Education intervention on early breast cancer detection	To increase the level of knowledge of early breast cancer detection 2011	Does not mention	Random probability sampling	352 women	Four training modules which included participatory techniques (group discussions and educational games) which lasted an hour for each group, once a week, for 3 months
14.	Gisela González Ruiz, Orlando Peralta González, Dayana Judith de la Rosa (2019)	SciELO	Impact of an educational intervention on breast cancer knowledge in Colombian women	Evaluation of the impact of educational intervention on knowledge of breast cancer in a group of adult women in Santa Marta.2020	Quasi-experimental	Non-probabilistic sample	96 women aged over of 20 years	Educational chats, teaching discussions and demonstrations on the self-breast-examination technique
15.	Amaya–Nieto M, Prado–Avendaño K, and Velásquez Carranza D (2015).	Google Scholar	Educational Intervention Efficacy in the level of knowledge about Breast Cancer in Tiwinsa-Puente Piedra Shanty Town Women	To determine the effectiveness of an educational intervention on the level of knowledge of breast cancer in women between 30 and 65 years of age created in Tiwinza Shanty Town, Puente Piedra in 2012	Pre-experimental	Probability sampling	61 women aged between 30 and 65 years old	Details of the intervention not referenced
16.	Scott R, Ramírez AF, Desten A and Soto O (2019)	SciELO	Educational intervention on breast cancer in women, University Polyclinic ‘Emilio Daudinot Bueno’, Guantánamo 2017-2018	To design an educational intervention aimed at raising the preparation on the subject in women from 18 and 60 years of the Family Office 12 of the University Polyclinic ‘Emilio Daudinot Bueno’	Prospective﻿	Availability	97 women 18–60 years	It was established in three phases. The first phase focused on the level of knowledge on the subject; the second stage involved the design and execution of the educational intervention. Three weekly educational sessions took place, lasting 50 minutes. The third stage entailed an evaluation of the results of the educational intervention
17.	Gisela González Ruiz, Orlando Peralta González, Dayana Judith de la Rosa (2019)	SciELO	Impact of an educational intervention on breast cancer knowledge in Colombian women	Evaluation of the impact of educational intervention on knowledge of breast cancer in a group of adult women in Santa Marta	Quasi-experimental	Non-probabilistic sample	96 women over the age of 20	3 sessions 15 workshops
18.	María Martínez Haro, Mª Dolores Quiñoz Gallardo, María Porta Sanfeliu (2015)	Google Scholar	Educational intervention about breast cancer prevention with health professionals within a hospital setting	Improve the knowledge of health professionals on prevention and health promotion related to the prevention and early diagnosis of breast cancer	Assessment by knowledge comparison pre and post an educational intervention	Does not mention	106 women/men over the age of 20 years (doctor, nurse, nursing assistant, non-health professionals)	3 theoretical-practical workshops with audiovisual media (video, Power Point presentation), lasting 2 hours every 15 days
19.	Ma. del Rocío Figueroa Varela Gloria Alejandra Vega Guerrero Raquel Rocío Hernández Pacheco (2020)	SciELO	Breast health self-care teaching strategies for young university students	To assess the effectiveness of three teaching strategies for self-care of breast health, in state university students in Nayarit Mexico	Quantitative and transversal, with scope descriptive	Non-probabilistic convenience sampling	63 students (12 men and 51 women)	Workshops based on: alternative strategy (4 hours combining various participative teaching methods) peer tutoring strategy (2 hours, informal presentation and participative educational workshop activities) traditional strategy (2 hours, informal presentation using a transmission/reception model)

Source: Ortega-Jiménez MC. 2021
